# In-vitro-cytotoxicity of cariostatic agents based on fluorides and lanthanide salts in L-929 fibroblasts

**DOI:** 10.1007/s00784-025-06429-8

**Published:** 2025-07-02

**Authors:** Tobias Akamp, Sandra Pohl, Konstantin J. Scholz, Philipp Sigl, Andreas Rosendahl, Melanie Wölflick, Florian Pielnhofer, Wolfgang Buchalla, Matthias Widbiller

**Affiliations:** 1 Department of Conservative Dentistry and Periodontology, University Hospital Regensburg, D-93053 Regensburg, Germany; 2 Department of Operative Dentistry and Periodontology, Center for Dental Medicine, Medical Center, Faculty of Medicine, University of Freiburg, D-79160 Freiburg, Germany; 3 Institute of Inorganic Chemistry, University of Regensburg, Regensburg, Germany

**Keywords:** Cariostatic agents, Toothpastes, Fluorides, Lanthanoid series elements, Cell survival, Cell death

## Abstract

**Objectives:**

Fluoride-based cariostatic agents are commonly used in dental products and are generally considered safe. However, ongoing discussions about potential adverse effects are driving research into alternative agents, such as lanthanide salts. This study aims to evaluate the cytotoxic effects of different cariostatic agents, including fluoride compounds (NaF, Na_2_PO_3_F, NH_4_F) and lanthanide salts (Ce(NO_3_)_3_, CeCl_3_, Sm(NO_3_)_3_, SmCl_3_).

**Materials and methods:**

Mouse fibroblasts (L-929) were cultured in Eagle’s Minimum Essential Medium supplemented with 5% fetal bovine serum and 1% penicillin-streptomycin. Cell viability was assessed via MTT assay after 24 h of exposure to test compounds at concentrations of 0.0128, 0.064, 0.32, 1.6, 8, 40, 200 and 1000 mM, and lactate dehydrogenase (LDH) release was quantified to assess membrane integrity. The accumulation of reactive oxygen species (ROS) was determined after 24 h. Data were analyzed by non-parametric methods at a significance level of α = 0.05 (Mann-Whitney *U* and Kruskal-Wallis tests).

**Results:**

Cell viability decreased significantly for NaCl and NaNO_3_ at 200 mM, and for NaF, NH_4_F, Ce(NO_3_)_3_, CeCl_3_, Sm(NO_3_)_3_ and SmCl_3_ at 1.6 mM, falling below 70% of control (*P* ≤ 0.0178). Similarly, LDH assays indicated a significant incline in cytotoxicity at a concentration of 200 mM for NaCl, NaNO_3_ and Na_2_PO_3_F, and at 8 mM for NaF, NH_4_F and all lanthanide compounds (*P* ≤ 0.0016). ROS quantification showed that NaF, NH_4_F, CeCl_3_, Sm(NO_3_)_3_ and SmCl_3_ induced oxidative stress at 1.6 mM with statistical significance (*P* ≤ 0.0065).

**Conclusions:**

Fluoride and lanthanide compounds exhibited similar in vitro biocompatibility, comparable to that of table salt.

**Clinical relevance:**

Both fluoride- and lanthanide-based cariostatic agents appear to pose a low biological risk to surrounding oral tissues when used at appropriate doses in dental products.

**Supplementary Information:**

The online version contains supplementary material available at 10.1007/s00784-025-06429-8.

## Introduction

Both caries and erosion are conditions that result in the loss of dental hard tissue. While caries is caused by bacteria that produce organic acids that demineralize enamel and dentin, erosive tooth wear is caused by chemical dissolution based on acids from other sources, such as sour beverages or citrus fruits [1, 2]. Preventive therapeutic approaches therefore aim to inhibit demineralization and support remineralization.

As early as 1939, J.F. Volker demonstrated that enamel powder became less acid soluble after treatment with sodium fluoride [3]. Since the market launch of the first fluoride-containing toothpaste in the 1950s, fluorides have been widely used as cariostatic agents to prevent demineralization or even remineralize affected dental hard tissues [4, 5]. Fluorides exert their cariostatic effect by lowering the solubility product of enamel hydroxyapatite, either through conversion to fluorapatite or via surface-limited ionic exchange within the crystal’s hydration layer or outer 1 to 2 nm [6–11]. A preventive effect could clinically be seen in terms of children and adolescents showing significantly lower DMFT (decayed, missing and filled teeth) scores when using fluoride toothpastes compared to non-fluoride toothpastes [12], which could be traced over the last 40 years in Germany [13].

While the caries-preventing effect of fluoride is now undisputed, and its effect against dental erosion has been shown [14], unjustified doubts about possible health risks of fluoride use have persisted for years. Numerous studies and everyday practice showed already that there are no adverse health effects associated with the regular use of fluoride-containing oral care products [10, 15]. When used correctly, these are not swallowed, but only come into direct contact with the epithelium of the oral cavity. Toothpaste has only been shown to be swallowed by infants up to 30 months of age [16, 17], which may be of concern as parents have been found to give toothpaste in doses exceeding recommended levels by a factor of 5.9 to 7.2 [18]. However, systemic effects such as fluorosis, which affects bones and teeth, or organ-related toxicity can only be considered a risk with enteral intake and at correspondingly high doses, e.g. ≥ 0.1 mg/kg body weight per day for children aged 1 to 8 years [19–22].

Nevertheless, the continued critical attitude, coupled with the fact that dental caries remains the most prevalent disease worldwide despite the widespread use of fluoride [23], continues to drive the ongoing development of new potentially cariostatic and anti-erosive agents. These include lanthanide compounds such as cerium salts, which were first considered as an alternative or addition to fluoride in 1999 but have not yet found their way into clinical practice [24, 25]. It has been demonstrated that cerium salts can accumulate on dental hard tissues, exhibit antibacterial activity against certain oral bacteria, and penetrate enamel. Due to their similarity to calcium, cerium ions may occupy calcium sites within the crystal structure of hydroxyapatite [24, 26–28]. This enhances the acid resistance of enamel and dentin, representing a promising cariostatic feature. Inhibition of demineralization and reduced lesion depth have been shown for cerium chloride and lanthanum, both alone and in combination with fluoride [24, 25, 29–31]. However, other studies have reported that while lanthanides may improve surface hardness, their effect on acid resistance is most evident when combined with fluoride [25, 32].

To be considered as a clinical alternative or adjunct to fluoride-based agents, lanthanide salts should not only offer advantages in terms of cariostatic performance, but should also be equivalent or superior in terms of biological safety, the latter argument being of particular interest for over-the-counter dental products used by patients at home. Since cariostatic agents are used topically in the oral cavity, oral tissues - particularly the gingival and mucosal layers - are of primary concern due to their direct exposure to dental care products. Despite the importance of this issue, few studies have addressed the cytotoxicity of both fluoride- and lanthanide-based products, and none have provided direct comparisons [33–37].

To investigate the biocompatibility of fluorides and lanthanide salts, this study aimed to evaluate the effects of NaF, Na_2_PO_3_F, NH_4_F, Ce(NO_3_)_3_, CeCl_3_, Sm(NO_3_)_3_ and SmCl_3_ on cell survival, metabolism and oxidative stress. The null hypothesis was that lanthanide compounds do not differ in cytotoxicity compared to fluoride-based products.

## Materials and methods

### Cell culture and chemicals

Sodium fluoride (≥ 99.0% purity), sodium nitrate (≥ 99.0% purity), sodium monofluorophosphate (95% purity), sodium chloride (≥ 99.0% purity), ammonium fluoride (≥ 99.99% purity) and cerium (III) chloride heptahydrate (≥ 98.0% purity) were purchased from Sigma-Aldrich (St. Louis, MO, USA). Samarium (III) chloride hexahydrate (99.9% purity), samarium (III) nitrate hexahydrate (99.9% purity) and cerium (III) nitrate hexahydrate (99.9% purity) were supplied by Chempur (Karlsruhe, Germany). In order to accurately weigh the chemicals, the amount of crystal water of the chemicals was determined by thermogravimetry (Mettler TG50 with Mettler Toledo STAR^e^ System) at the Institute of Inorganic Chemistry at the University of Regensburg.

Mouse fibroblasts of the L-929 line (NCTC clone 929 [L cell, L-929, derivative of strain L], CCL-1) were obtained from the American Type Culture Collection (ATCC; Manassas, VA, USA) and used for all experiments according to ISO 10993-5. L-929 cells are sourced from mouse subcutaneous connective tissue and were cultured in Eagle’s Minimum Essential Medium (MEM; Sigma-Aldrich) supplemented with 5% fetal bovine serum and 1% penicillin-streptomycin (Sigma-Aldrich). Experiments were performed in 96-well culture plates with 10,000 cells (passage 10) per well. Cell number was determined using a TC-20 cell counter after trypan blue staining (Bio-Rad, Hercules, CA, USA). After 24 h of cell attachment, the culture medium was replaced with medium containing test material solutions at varying concentrations (0.0128, 0.064, 0.32, 1.6, 8, 40, 200 and 1000 mM). For control, cells were cultured in medium without supplements.

### MTT assay

Cell viability was determined by the MTT assay after 24 h of exposure to salt solutions. After removal of the culture medium, cells were incubated with 100 µl per well of MTT solution (0.5 mg/mL; 3-(4,5-dimethyl-2-thiazolyl)-2,5-diphenyl-2 H-tetrazoliumbromid; Sigma-Aldrich) in MEM at 37 °C in 5% CO_2_ for 2 h. The converted dye was dissolved in 100 µl DMSO (dimethyl sulfoxide; Merck Millipore, Burlington, MA, USA) per well and incubated at 37 °C in 5% CO_2_ with continuous shaking for 20 min. The absorbance of MTT-derived formazan was measured on a microplate reader at λ = 540 nm (Infinite 200; Tecan, Männedorf, Switzerland). Three experiments were performed each with eight replicates (*n* = 24). Medians and interquartile range (25th-75th percentiles) were calculated and presented relative to the untreated control, which was set at 100%.

### LDH assay

In analogy to the MTT assay, the amount of lactate dehydrogenase (LDH) released into the culture medium, reflecting cell membrane integrity was quantified by a colorimetric assay (CytoTox 96 Non-Radioactive Cytotoxicity Assay; Promega, Madison, WI, USA) after 24 h. Photometric measurement was performed on a microplate reader at λ = 450 nm (Infinite 200; Tecan, Männedorf, Switzerland). Experiments were performed in eight replicates and repeated twice (*n* = 24). According to the manufacturer’s instructions, cytotoxicity was calculated as a percentage of the maximum expected LDH release from fully lysed cells. This maximum release was determined by complete lysis of control cells using 10 µL of 10× lysis solution added to 100 µL of cell suspension, 45 min before the addition of CytoTox 96 reagent. Medians and interquartile range (25th-75th percentiles) were calculated based on LDH release from the experimental and lysis control groups.

### Light microscopy

To gain insight into the changes in cell morphology upon treatment with different concentrations of fluorides and lanthanide salts, images of the cultures were taken using an inverted light microscope and a corresponding camera system (Axio Vert.A1; Carl Zeiss Microscopy GmbH, Jena, Germany).

### Cell number assay

After 24 h of treatment, cell numbers were determined using the CyQUANT Cell Proliferation Assay Kit from Invitrogen (Waltham, MA, USA) according to the manufacturer’s instructions. Fluorescence intensity, which reflects the amount of CyQUANT GR dye bound to cellular nucleic acid, was measured using a microplate reader with an excitation at λ = 485 nm and emission at λ = 535 nm. Three experiments were conducted with seven replicates (*n* = 21) and results were normalized to the untreated control.

### ROS assay

The accumulation of reactive oxygen species (ROS) in cells was determined after 24 h of exposure using a fluorescence assay with the cell-permeable reagent 2’,7’-dichlorofluorescein diacetate (DCFDA). The cell culture medium was removed and cells were washed with 250 µl phosphate-buffered saline (Sigma-Aldrich), followed by incubation with 100 µl DCFDA solution (20 mM; Merck Millipore) for 30 minutes. Cellular esterases deacetylate DCFDA to a non-fluorescent compound, which is subsequently oxidized by present ROS to the highly fluorescent 2’,7’dichlorofluorescein (DCF). The resulting fluorescence intensity was measured on a microplate reader with excitation and emission at λ = 485 nm and λ = 535 nm, respectively (Infinite 200; Tecan). Each experiment was performed three times with seven replicates (*n* = 21). Medians and interquartile range (25th-75th percentiles) were normalized to the control and related to the corresponding cell number.

### Statistical analysis

Data were assessed for normality, followed by analysis using non-parametric methods at a significance level of α = 0.05. Pairwise comparisons between test and control groups were performed using the Mann-Whitney U test. The Kruskal-Wallis test followed by Dunn’s multiple comparison test was used to compare various concentrations with untreated control.

All statistical calculations were performed using GraphPad Prism 9 (GraphPad Software; La Jolla, CA, USA). Results from multiple replicates were pooled, normalized to the untreated control and reported as medians with interquartile range (25th-75th percentiles). Significantly different results (*P* ≤ 0.05) are indicated with an asterisk and test results are provided in Supplementary File.

## Results

### Cell viability

**Fig. 1:**
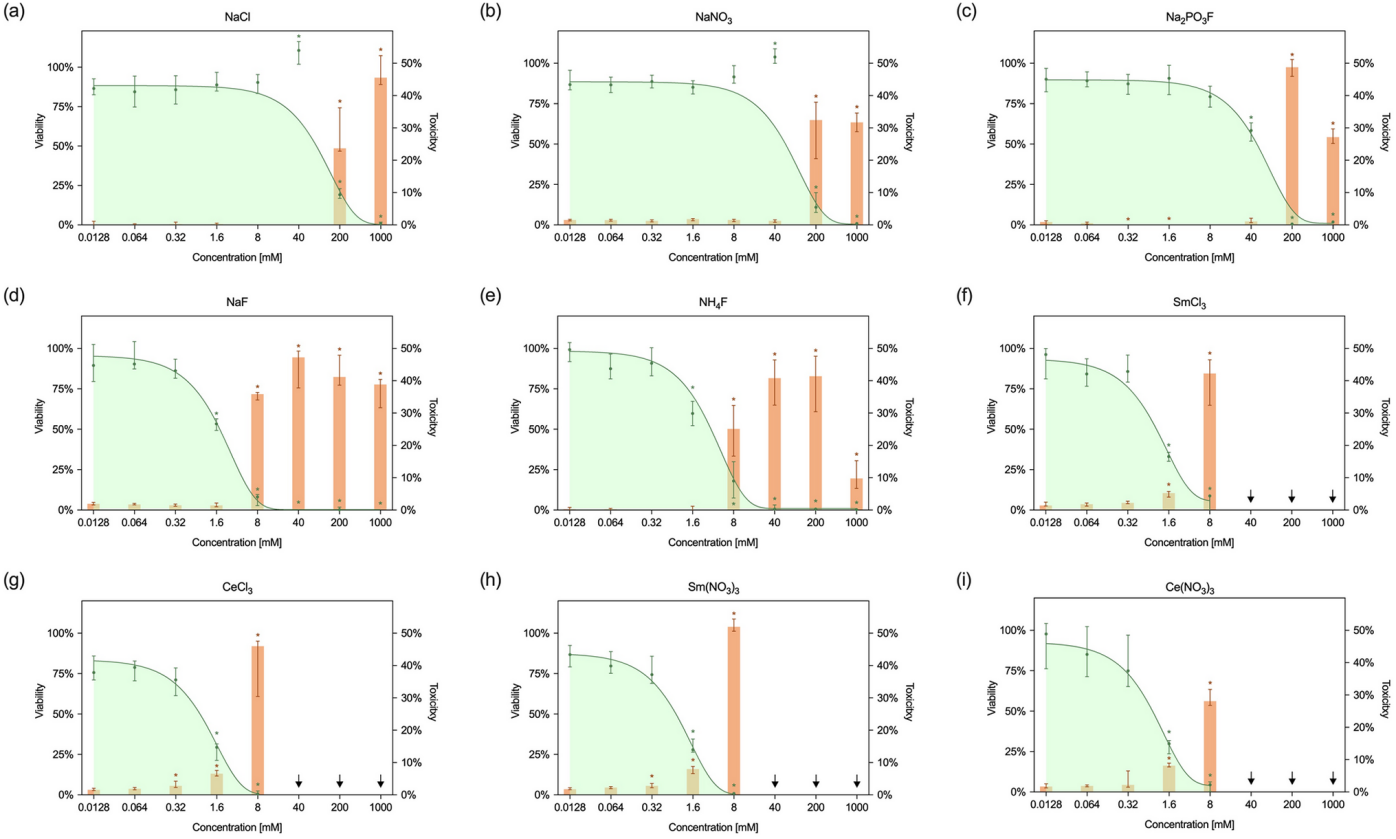
Cell viability (green, left y-axis) and cytotoxicity (orange, right y-axis) assessed by MTT and LDH assays, respectively. Viability is expressed relative to the untreated control, toxicity relative to the lysis control (median: 1.16785; interquartile range: 0.9471–1.32915). Data are presented as medians with interquartile range (25th-75th percentiles). Asterisks denote significant differences compared to the untreated control (*P* ≤ 0.05). Concentrations marked with downward arrows were excluded due to visible precipitation

According to ISO standard 10993-5, a viability of less than 70% compared to the control indicates a potential cytotoxicity for both the material and the concentration tested. Analysis of L-929 fibroblast cells treated with control compounds, namely sodium chloride and sodium nitrate, showed a significant decrease in cell viability below 70% when exposed to concentrations of 200 mM (*P* < 0.0001) (Fig. 1).

Experimental evaluation with fluorides specified a cytotoxic potential with statistical significance at concentrations of 40 mM for Na_2_PO_3_F and 1.6 mM for NaF and NH_4_F (*P* ≤ 0.0178) (Fig. 1). In particular, a significant reduction in viable cells was observed following exposure to the lanthanide salts Ce(NO_3_)_3_, CeCl_3_, Sm(NO_3_)_3_, and SmCl_3_ at concentrations exceeding 1.6 mM (*P* < 0.0001). However, viability data for lanthanide salt concentrations above 8 mM were not analysed because they exceeded their solubility limit and formed precipitates (Fig. 1).

### Membrane integrity

The quantitative assessment of LDH as a parameter of cytotoxicity is shown on the right axis in Fig. 1. For NaCl and NaNO_3_, no LDH release was detected at concentrations up to 40 mM. However, at higher concentrations, a significant cytotoxic effect of up to 45% was observed compared to untreated control cells (*P* < 0.0001) (Fig. 1). Na_2_PO_3_F showed similar behaviour, inducing approximately 50% cytotoxicity at a concentration of 200 mM with statistical significance (*P* < 0.0001). Conversely, the application of NaF and NH_4_F resulted in significantly elevated LDH levels at concentrations at 8 mM (*P* ≤ 0.0016), corresponding to toxicity levels also up to 50% (Fig. 1). In the case of lanthanide salts, Ce(NO_3_)_3_, CeCl_3_, Sm(NO_3_)_3_, and SmCl_3_, a marginal but significant increase in LDH release was noticed at a concentration of 1.6 mM (*P* < 0.0001), while considerable toxicity levels were observed at concentrations of 8 mM (*P* < 0.0001). Again, lanthanide solutions above 8 mM were not analysed because they exceeded the solubility limit (Fig. 1).

**Fig. 2:**
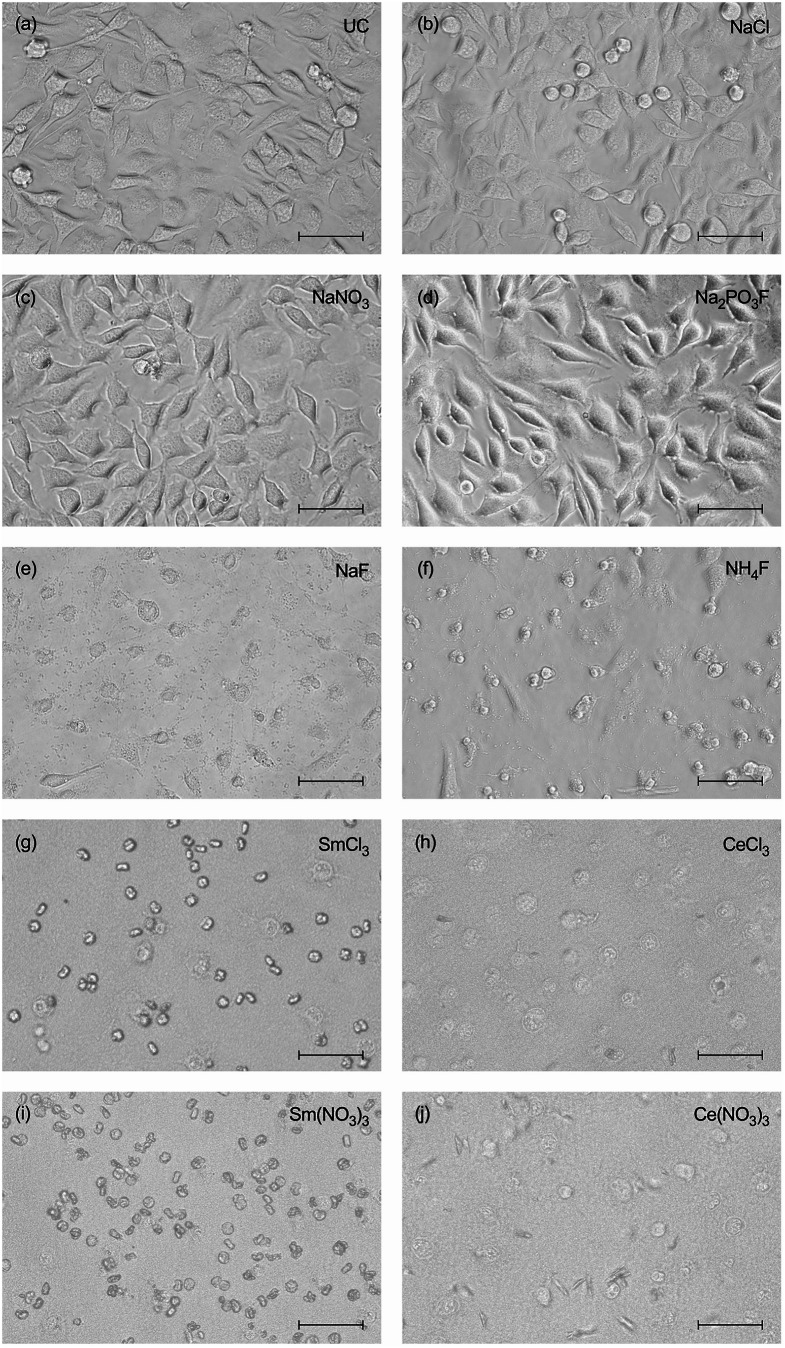
Light microscopy images of L-929 cells cultured for 24 h without (**a**) or with (**b**-**j**) 8 mM test compounds. While untreated cells (**a**), NaCl (**b**), NaNO_3_ (**c**) and Na_2_PO_3_F (**d**) showed normal morphology, cytotoxic changes were observed for NaF (**e**), NH_4_F (**f**), SmCl_3_ (**g**), CeCl_3_ (**h**), Sm(NO_3_)_3_ (**i**) and Ce(NO_3_)_3_ (**j**). Scale bar: 50 μm

### Cell morphology

Light microscopy revealed morphological changes in cells exposed to test compounds compared to untreated cells. Microscopic images of the untreated control showed an adherent monolayer of L-929 cells with a spindle-shaped, fibroblastic morphology (Fig. 2a). Figures 2b-j show cells incubated with 8 mM of the compounds tested. Cell death is characterized by spherical shape and loss of adherence, which was clearly observed for NaF, NH_4_F, Ce(NO_3_)_3_, CeCl_3_, Sm(NO_3_)_3_ and SmCl_3_ (Fig. 2e-j). However, cells exposed to Na_2_PO_3_F, NaCl and NaNO_3_ revealed no signs of cytotoxicity at 8 mM (Fig. 2b-d), but at higher concentrations (data not shown).

### Oxidative stress response

**Fig. 3:**
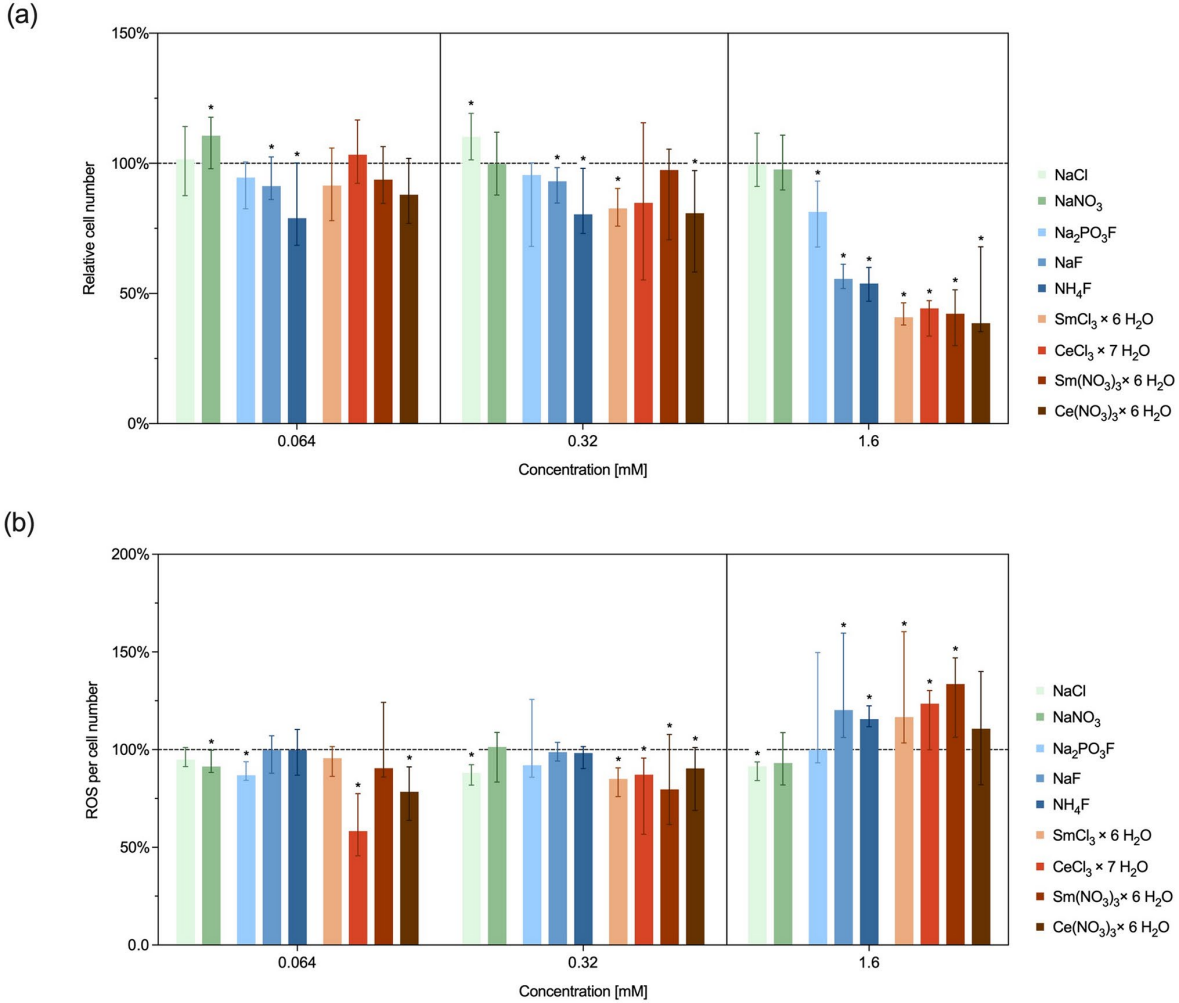
Cell number (**a**) and reactive oxygen species production (**b**) in L-929 cells after 24 h exposure to test compounds. The dotted line indicates the median for the untreated control. Data are presented as medians with interquartile range (25-75th percentiles). Asterisks denote significant differences compared to the untreated control (*P* ≤ 0.05)

Analyses of ROS levels were performed in a concentration range of interest (0.0064 to 1.6 mM) defined on the basis of viability and toxicity parameters. ROS levels are indicative of cellular stress and are related to the corresponding cell counts (Fig. 3).

While only NaF and NH_4_F caused a significant reduction (*P* ≤ 0.0382) in cell number at 0.064 mM (Fig. 3a), a decrease with statistical significance was observed at 0.32 mM for NaF, NH_4_F, SmCl_3_ and Ce(NO_3_)_3_ (*P* ≤ 0.0100). At 1.6 mM, Na_2_PO_3_F significantly reduced cell numbers to approximately 80% of the control (*P* = 0.0044), whereas NaF (56%), NH_4_F (54%), and the lanthanide salts (39 to 44%) led to a more pronounced decline (*P* < 0.0001). In contrast, NaCl and NaNO_3_ did not significantly affect cell counts (*P* > 0.5792).

In general, test material concentrations below 1.6 mM resulted in ROS levels equal to or slightly lower than those of untreated cells (Fig. 3b). For NaF, NH_4_F, CeCl_3_, Sm(NO_3_)_3_ and SmCl_3_, significantly increased ROS levels in the cells could be detected at salt concentrations of 1.6 mM (*P* ≤ 0.0065). In contrast, Na_2_PO_3_F and Ce(NO_3_)_3_ showed no significant increase in ROS at 1.6 mM (*P* > 0.2941).

## Discussion

Lanthanide compounds have shown promising anti-caries potential in preclinical studies, suggesting that they may serve as a viable alternative or adjunct to fluoride application strategies [30, 35]. They appear to be advantageous due to their physicochemical properties, particularly in enhancing remineralization and reducing demineralization of dental hard tissues through optimized molecular lattice integration [30, 38]. For example, cerium ions can adhere to and accumulate on the enamel surface and have been observed to integrate into synthesized hydroxyapatite crystal lattice [26, 27, 38]. Recent studies have further demonstrated that cerium and samarium nitrates not only accumulate on dentin or accumulate on and penetrate into sound enamel surfaces, but also exhibit antibacterial activity in planktonic bacterial cultures [27, 28]. Given their potential for clinical use in caries prevention and ongoing insecurities regarding the biocompatibility of oral agents, a comprehensive biological evaluation needs to be conducted before proceeding with clinical trials.

There are two aspects to consider when assessing biocompatibility or toxicity: the cytotoxic properties of substances affecting oral tissues during local application and systemic toxicity following absorption and metabolization by the organism.

The local cytotoxic effects of fluoride are associated with oxidative stress, cell cycle arrest and apoptosis, resulting in nuclear condensation, nuclear membrane rupture, mitochondrial vacuolization, fragmentation and mitochondrial fission [39]. When fluoride is absorbed systemically at high levels, these effects can cause mineralization disorders, namely fluorosis, or irreversible damage to the target organs brain, liver and kidney [40–42]. With regard to the systemic effects of rare earths, some toxicity to lung, liver and brain tissues has been observed, although it is reported to be less severe than that of fluoride [43, 44]. In particular, lanthanum and cerium have been shown to be less toxic than fluoride, with a reduced tendency to accumulate in the liver and kidneys [44].

In populations consuming 0.08 to 0.12 mg fluoride per kg body weight per day, the prevalence of moderate dental fluorosis of permanent teeth is reported to be less than 5% [22]. Studies indicate that reducing fluoride levels in drinking water to 0.06 to 0.08 parts per million (ppm) significantly reduces the incidence of fluorosis [45]. This suggests that fluorides pose a minimal systemic risk if individuals consume the recommended maximum daily intake. The probable toxic dose (PTD) of fluoride is approximately 5 mg fluoride per kg body weight per day, while the certainly toxic dose (CTD) for humans is 32 to 64 mg fluoride per kg body weight per day [46]. In comparison, the mean lethal doses of, for example, cerium nitrate (4200 mg/kg bw; rat) and samarium nitrate (2900 mg/kg bw; rat) exceed even the mean human lethal dose of sodium chloride (2690 mg/kg bw) [33, 34].

However, since fluoride products or lanthanide preparations are used locally as cariostatic agents and are not intended to be absorbed systemically, the focus of the studies was not on systemic toxicity but on direct toxicity to biologically exposed tissues. In addition to fluoride (NaF, NH_4_F, Na_2_PO_3_F) and lanthanide compounds (Ce(NO_3_)_3_, CeCl_3_, Sm(NO_3_)_3_, and SmCl_3_), NaCl and NaNO_3_ were used as control substances in the present study. Although these do not play a direct role in dentistry, they are used in the food sector as flavorings or preservatives, so a reasonable interpretation and realistic classification of the hazard potential of the substances studied is possible. In addition, both salts contain anions and cations that are also part of the fluoride and lanthanide compounds and may allow conclusions about the specific effect of individual anions or cations.

Although there are individual studies on the in-vitro-cytotoxicity of fluorides and cerium salts [35–37, 47, 48], they do not meet international standards. ISO standard 10993-5 specifies test methods for the evaluation of dental materials, including fluoride varnishes, and is used to ensure that materials are safe and effective before they are approved for clinical use. Cariostatic agents are typically water-soluble or incorporated into chemically curing matrices designed to prolong contact with enamel and dentin. In addition to dental hard tissues, these agents also come into direct contact with the gingiva and oral mucosa. To evaluate their biocompatibility in vitro, cells were therefore directly exposed to fluoride and lanthanide salts dissolved in culture medium for 24 h.

Experimental parameters such as test methods, exposure conditions, biological endpoints and cell lines are critical in the assessment of cytotoxicity. Continuous cell lines such as L-929 are commonly used and endorsed by ISO 10993-5 for dental product testing due to their manageable culture conditions. While cells isolated from target oral tissues may provide more accurate toxicological data, primary cells tend to be less sensitive and high donor variability may limit comparability between studies [49]. Therefore, L-929 fibroblasts were chosen for this study. As an immortalized and commercially available cell line, they allow reproducible experiments and facilitate comparison of results with other studies.

Cytotoxic agents can typically induce both apoptosis and necrosis, two distinct but related forms of cell death. Apoptosis is a tightly regulated, caspase-dependent process triggered by intracellular damage sensors or death-receptor activation. It features cell shrinkage, chromatin condensation, membrane blebbing and the formation of apoptotic bodies, with membrane permeabilization releasing small amounts of LDH [50]. In contrast, necrosis is an uncontrolled, lytic form of cell death induced by severe external insults, such as radiation, heat, chemicals or hypoxia [51]. It involves the upregulation of pro-inflammatory proteins and chemokines, cytoplasmic swelling, nuclear disintegration and the plasma membrane rupture [51], liberating intracellular components and provoking inflammation [52, 53]. Under certain conditions, such as high irritant concentrations or long exposure times, apoptotic cells may fail to complete programmed death and instead undergo necrosis. Although many studies have examined these mechanisms in isolation, comprehensive biocompatibility testing must comply with international standards [35–37, 47, 48]. Multiple cytotoxicity endpoints (cell viability, LDH release, proliferation, and oxidative stress) were investigated in this study to capture the full spectrum of cellular responses.

A decrease in cellular metabolism is recognized as an initial cytotoxic response in cells and is regarded as a surrogate for cell viability. Even minor shifts in metabolic activity can lead to significant fluctuations in MTT levels, enabling the detection of cell stress upon exposure to a cytotoxic compound [54]. The criteria for cytotoxicity defined in ISO standard 10993-5 define a reduction in cell metabolism of more than 30% as cytotoxic [55]. Results of this study show an influence of all test materials on cell viability in a concentration-dependent manner.

For all lanthanide salts, concentrations of 1.6 mM and above affected cell viability of more than 30% and thus representing cytotoxicity. However, a study by Schmidlin et al. found enhanced proliferation, differentiation, viability and migration of fibroblasts and depressive effects on osteoblasts after exposure to CeCl_3_ for 10 s and further incubation [35]. While up to 100 mM Ce^3+^ still led to an increase in viability of fibroblasts, a decrease in cell viability for Cerium concentrations of 50 mM or higher was observed for osteoblasts [35]. In the present study, a maximum concentration of 8 mM was utilized for all lanthanide compounds. Higher concentrations exhibited limited solubility, resulting in pH levels around 2 upon dilution with media, which would have been harmful for cell cultures for various reasons. These aspects and the differences in cell types, concentrations and exposure times limit direct comparability between these studies.

Likewise, NaF and NH_4_F caused a reduction in cell metabolism of more than 30% at 1.6 mM and above, however, Na_2_PO_3_F appeared to be cytotoxic only at 40 mM and above. This may be due to the different chemical bonding types of the fluoride compounds. While NaF and NH_4_F are ionic compounds that dissolve easily in aqueous solutions, in Na_2_PO_3_F fluoride is covalently bonded to phosphorous and requires hydrolysis to release the fluoride ion. In the oral environment, Na_2_PO_3_F is adsorbed onto the enamel surface, where it may be hydrolyzed by salivary components or plaque enzymes [56, 57], releasing fluoride ions that may potentially diffuse into the enamel [58, 59]. However, the lower toxicity of Na_2_PO_3_F, due to its slower fluoride ion release through hydrolysis, may, under certain circumstances, also be associated with reduced cariostatic effectiveness compared to sodium fluoride [60], which releases fluoride ions immediately upon dissolution [61, 62]. These findings are consistent with those by López-García et al., who reported that fluoride varnishes containing NaF or NH_4_F were cytotoxic to human gingival fibroblasts [63]. Other studies found that fluoride concentrations between 0.5 and 12 mM resulted in a reduction of mitochondrial activity in human dental pulp cells [37] and human oral mucosal fibroblasts [47].

The careful selection of test materials for this study, which dissociate in aqueous solution releasing anions and cations, provides insight into the cytotoxicity of individual ions. Controls were used to exclude the possibility that the cation Na^+^ or the anions Cl^−^ and NO_3_^−^ were causing cytotoxicity. These controls, as well as Na_2_PO_3_F, caused a decrease in cell viability at concentrations ≥ 200 mM, which is a significantly higher threshold than 1.6 mM for NaF, NH_4_F and all lanthanide salts. This difference may be due to the specific effects of both lanthanide and fluoride ions on cells. Lanthanides are known to affect oxidative metabolism and induce apoptosis [64–66], whereas fluoride can disrupt enzyme activity and alter signaling pathways [67, 68]. In this respect, the same effects have not been described for Na^+^, Cl^−^ and NO_3_^−^. Therefore, the cytotoxic effects of lanthanide and fluorides on cell viability can be attributed primarily to cerium, samarium, and fluoride ions.

Escalating extracellular stressors can cause damage to the cell plasma membrane [69]. LDH is a stable enzyme present in the cytoplasm that is rapidly released into the cell culture medium upon membrane damage, thus, serving as a hallmark of cytotoxicity [70]. In this study, all lanthanide salts and ionically bound fluoride salts showed increased LDH release at concentrations of 8 mM, whereas controls and Na_2_PO_3_F showed increased release at 200 mM. It is conclusive that in the experiments, a decrease in cell viability was always accompanied by a concentration-dependent increase in LDH release, indicating toxicity. The results align with previous research indicating an increased membrane permeability at concentrations of 2 or 4 mM NaF in fibroblasts and osteocytes [47, 71]. LDH release was not yet tested for lanthanide salts, which is why our results cannot be set in context with other studies.

ROS are highly reactive compounds, often radicals, that are both toxic to pathogens and act as signaling molecules in inflammatory processes, whereby their influence on the immune response is complex and can be either pro- or anti-inflammatory depending on the context [53, 72]. Increased abundance of reactive oxygen species is commonly observed during inflammatory processes. ROS activate pro-inflammatory pathways like NF-κB and MAPKs, leading to cytokine release and immune cell recruitment, while also triggering inflammasomes to enhance inflammation [73–75]. Except for Na_2_PO_3_F, all fluorides and lanthanide salts were found to induce oxidative stress in mouse fibroblasts at concentrations of 1.6 mM and higher. Previous studies also found increased ROS levels in fibroblasts exposed to lanthanide and ionically bound fluoride salts at 1.6 mM, whereas NaCl and NaNO_3_ had no negative effect. It was reported that NaF induces ROS production to induce apoptosis by activating ROS-dependent pathways such as NF-κB or p53/DR5 signaling pathway [76, 77]. Similarly, lanthanides were described to induce oxidative injury in neurons and astrocytes by increasing ROS production and lipid peroxidation and decreasing the activity of key enzymes such as superoxide dismutase, catalase, ascorbic acid, and glutathione peroxidases, ultimately leading to apoptosis [78, 79].

Overall, concentration-dependent effects were observed, leading to a reduction in cell viability, membrane damage and intracellular stress with cell death above a certain threshold, which does not appear to be clinically critical. In the interpretation of in-vitro-cytotoxicity test results, it’s important to be aware that both control and test substances may be present at much higher concentrations in the clinical setting. However, they are typically diluted by body fluids or beverages and, unlike in vitro, interact with tissues and cells for only a short time. For example, consumption of table salt (NaCl) involves much higher concentrations, and fluoride in toothpaste can initially reach levels up to 50 times higher than, for instance, a 1.6 mM solution, yet no toxic reactions are observed clinically. However, the effects of ionic fluorides (NaF, NH_4_F) and lanthanides (Ce(NO_3_)_3_, CeCl_3_, Sm(NO_3_)_3_, SmCl_3_) were comparable. In contrast, Na_2_PO_3_F showed a cytotoxic effect comparable to NaCl and NaNO_3_ only at higher concentrations with the covalently bound fluoride. Based on the data, the null hypothesis of no difference in cytotoxicity between lanthanide and fluoride compounds cannot be rejected.

## Conclusion

In conclusion, both fluoride and lanthanide compounds demonstrated similar biocompatibility in vitro, with cytotoxic effects such as reduced viability, oxidative stress, and membrane damage occurring with increasing concentration. Given the extensive clinical history of fluoride use and the limited, low-dose exposure associated with lanthanide preparations in clinical settings, no significant biological risk to oral tissues is anticipated from the introduction of lanthanides. This suggests that lanthanide-based treatments may offer a safe alternative or complement to traditional fluoride therapies in dental care.

## Electronic supplementary material

Below is the link to the electronic supplementary material.

Supplementary Material 1

## Data Availability

The datasets generated during and/or analysed during the current study are available from the corresponding author on reasonable request.
