# Supplementary material for: In-vitro-cytotoxicity of cariostatic agents based on fluorides and lanthanide salts in L-929 fibroblasts
Source: Clin Oral Investig. 2025 Jul 2;29(7):366. doi: 10.1007/s00784-025-06429-8 (PMC12213984; doi:10.1007/s00784-025-06429-8)
Supplement: Supplementary file 1 — Supplementary Material 1 [file 784_2025_6429_MOESM1_ESM.pdf]

Supplementary File

In-vitro-cytotoxicity of cariostatic agents based on fluorides and lanthanide salts in L-929 fibroblasts

Tobias Akamp[1], Sandra Pohl[1*], Konstantin J. Scholz[1,2], Philipp Sigl[1], Andreas Rosendahl[1], Melanie Wölflick[1], Florian Pielnhofer[3], Wolfgang Buchalla[1], Matthias Widbiller[1]*

[1] Department of Conservative Dentistry and Periodontology, University Hospital Regensburg, Regensburg, Germany
[2] Department of Operative Dentistry and Periodontology, Center for Dental Medicine, Medical Center, University of Freiburg, Faculty of Medicine, University of Freiburg, Germany
[3] Institute of Inorganic Chemistry, University of Regensburg, Regensburg
[*] These authors contributed equally to this work

Corresponding author

Prof. Dr. Matthias Widbiller
Department of Conservative Dentistry and Periodontology
University Hospital Regensburg
D-03053 Regensburg
Germany
E-Mail: matthias@widbiller.de

Supplementary Table 1 Cell viability, *P*-values from Dunn's multiple comparison test following Kruskal-Wallis tests comparing each compound's concentrations with the untreated control.

Concentration [mM]	Compound								
	NaCl	$NaNO_3$	Na_2PO_3F	NaF	NH_4F	$SmCl_3$	$CeCl_3$	$Sm(NO_3)_3$	$Ce(NO_3)_3$
0.064	>0.9999	>0.9999	>0.9999	>0.9999	>0.9999	0.6431	>0.9999	>0.9999	>0.9999
0.32	>0.9999	>0.9999	>0.9999	>0.9999	>0.9999	0.9812	0.7818	0.1225	0.3946
1.6	>0.9999	>0.9999	>0.9999	0.0178	0.0017	<0.0001	<0.0001	<0.0001	<0.0001
8	>0.9999	>0.9999	0.1496	<0.0001	<0.0001	<0.0001	<0.0001	<0.0001	<0.0001
40	0.0011	0.0004	<0.0001	<0.0001	<0.0001				
200	<0.0001	<0.0001	<0.0001	<0.0001	<0.0001				
1000	<0.0001	<0.0001	<0.0001	<0.0001	<0.0001				

Supplementary Table 2 Membrane integrity, *P*-values from Dunn's multiple comparison test following Kruskal-Wallis tests comparing each compound's concentrations with the untreated control.

Concentration [mM]	Compound								
	NaCl	$NaNO_3$	Na_2PO_3F	NaF	NH_4F	$SmCl_3$	$CeCl_3$	$Sm(NO_3)_3$	$Ce(NO_3)_3$
0.064	>0.9999	>0.9999	>0.9999	>0.9999	>0.9999	>0.9999	>0.9999	0.5765	>0.9999
0.32	>0.9999	>0.9999	0.0257	0.8645	>0.9999	0.4729	0.0057	0.0120	0.7288
1.6	>0.9999	>0.9999	0.0023	>0.9999	>0.9999	<0.0001	<0.0001	<0.0001	<0.0001
8	0.6087	>0.9999	0.2812	0.0016	<0.0001	<0.0001	<0.0001	<0.0001	<0.0001
40	0.7010	0.5486	>0.9999	<0.0001	<0.0001				
200	<0.0001	<0.0001	<0.0001	<0.0001	<0.0001				
1000	<0.0001	<0.0001	0.0006	<0.0001	0.0026				

Supplementary Table 3 Cell number, *P*-values from pairwise Mann-Whitney *U* tests comparing the compounds at each concentration with the untreated control.

Concentration [mM]	Compound								
	NaCl	$NaNO_3$	Na_2PO_3F	NaF	NH_4F	$SmCl_3$	$CeCl_3$	$Sm(NO_3)_3$	$Ce(NO_3)_3$
0.064	0.4068	0.0336	0.4697	0.0382	0.0067	0.3088	0.0736	0.4458	0.1079
0.32	0.0084	0.8813	0.1930	0.0100	0.0026	0.0047	0.1046	0.4688	0.0037
1.6	0.5792	0.8034	0.0044	<0.0001	<0.0001	<0.0001	<0.0001	<0.0001	<0.0001

Supplementary Table 4 Reactive oxygen species (ROS), *P*-values from pairwise Mann-Whitney *U* tests comparing the compounds at each concentration with the untreated control.

Concentration [mM]	Compound								
	NaCl	$NaNO_3$	Na_2PO_3F	NaF	NH_4F	$SmCl_3$	$CeCl_3$	$Sm(NO_3)_3$	$Ce(NO_3)_3$
0.064	0.1762	0.0209	0.0015	0.6904	0.7842	0.0927	<0.0001	0.0927	<0.0001
0.32	<0.0001	0.7842	0.1530	0.5170	0.1762	<0.0001	0.0013	0.0217	0.0160
1.6	0.0002	0.3302	0.9010	<0.0001	<0.0001	0.0002	0.0065	0.0025	0.2941
